# Enhancing β-Carotene Production in *Escherichia coli* by Perturbing Central Carbon Metabolism and Improving the NADPH Supply

**DOI:** 10.3389/fbioe.2020.00585

**Published:** 2020-06-09

**Authors:** Yuanqing Wu, Panpan Yan, Yang Li, Xuewei Liu, Zhiwen Wang, Tao Chen, Xueming Zhao

**Affiliations:** ^1^Synthetic Biology and Key Laboratory of Systems Bioengineering (Ministry of Education), Tianjin, China; ^2^SynBio Research Platform, Collaborative Innovation Center of Chemical Science and Engineering (Tianjin), School of Chemical Engineering and Technology, Tianjin University, Tianjin, China; ^3^College of Life Science, Shihezi University, Shihezi, China

**Keywords:** *Escherichia coli*, metabolic engineering, β-carotene, phosphotransferase system inactivation, NADPH supply

## Abstract

Beta (β)-carotene (C_40_H_56_; a provitamin) is a particularly important carotenoid for human health. Many studies have focused on engineering *Escherichia coli* as an efficient heterologous producer of β-carotene. Moreover, several strains with potential for use in the industrial production of this provitamin have already been constructed via different metabolic engineering strategies. In this study, we aimed to improve the β-carotene-producing capacity of our previously engineered *E. coli* strain ZF43Δ*gdhA* through further gene deletion and metabolic pathway manipulations. Deletion of the *zwf* gene increased the resultant strain's β-carotene production and content by 5.1 and 32.5%, respectively, relative to the values of strain ZF43Δ*gdhA*, but decreased the biomass by 26.2%. Deletion of the *ptsHIcrr* operon further increased the β-carotene production titer from 122.0 to 197.4 mg/L, but the provitamin content was decreased. Subsequently, comparative transcriptomic analysis was used to explore the dynamic transcriptional responses of the strains to the blockade of the pentose phosphate pathway and inactivation of the phosphotransferase system. Lastly, based on the analyses of comparative transcriptome and reduction cofactor, several strategies to increase the NADPH supply were evaluated for enhancement of the β-carotene content. The combination of *yjgB* gene deletion and *nadK* overexpression led to increased β-carotene production and content. The best strain, ECW4/p5C-*nadK*, produced 266.4 mg/L of β-carotene in flask culture and 2,579.1 mg/L in a 5-L bioreactor. The latter value is the highest reported from production via the methylerythritol phosphate pathway in *E*. *coli*. Although the strategies applied is routine in this study, the combinations reported were first implemented, are simple but efficient and will be helpful for the production of many other natural products, especially isoprenoids. Importantly, we demonstrated that the use of the methylerythritol phosphate pathway alone for efficient β-carotene biosynthesis could be achieved via appropriate modifications of the cell metabolic functions.

## Introduction

Carotenoids, which are natural pigments that provide the yellow to pink to red colors in various species and are widespread in plants, animals, and microorganisms, have a variety of biological functions (Alcaíno et al., 2016). Beta (β)-carotene (C40; also known as provitamin A) is a particularly important carotenoid for human health (Kagechika and Shudo, 2005). Currently, more than 90% of commercial β-carotene is produced by chemical synthesis (Wu et al., 2017), which has low bioactivity since it has one dominant isomer and none of the other bioactive isomers and carotenoids associated with natural β-carotene (Gong and Bassi, 2016), thus, it is less effective and only used to animal feed and colorant. Furthermore, chemically synthesized β-carotene is not considered as safe as its natural counterpart (Woutersen et al., 1999).

**Figure 1:**
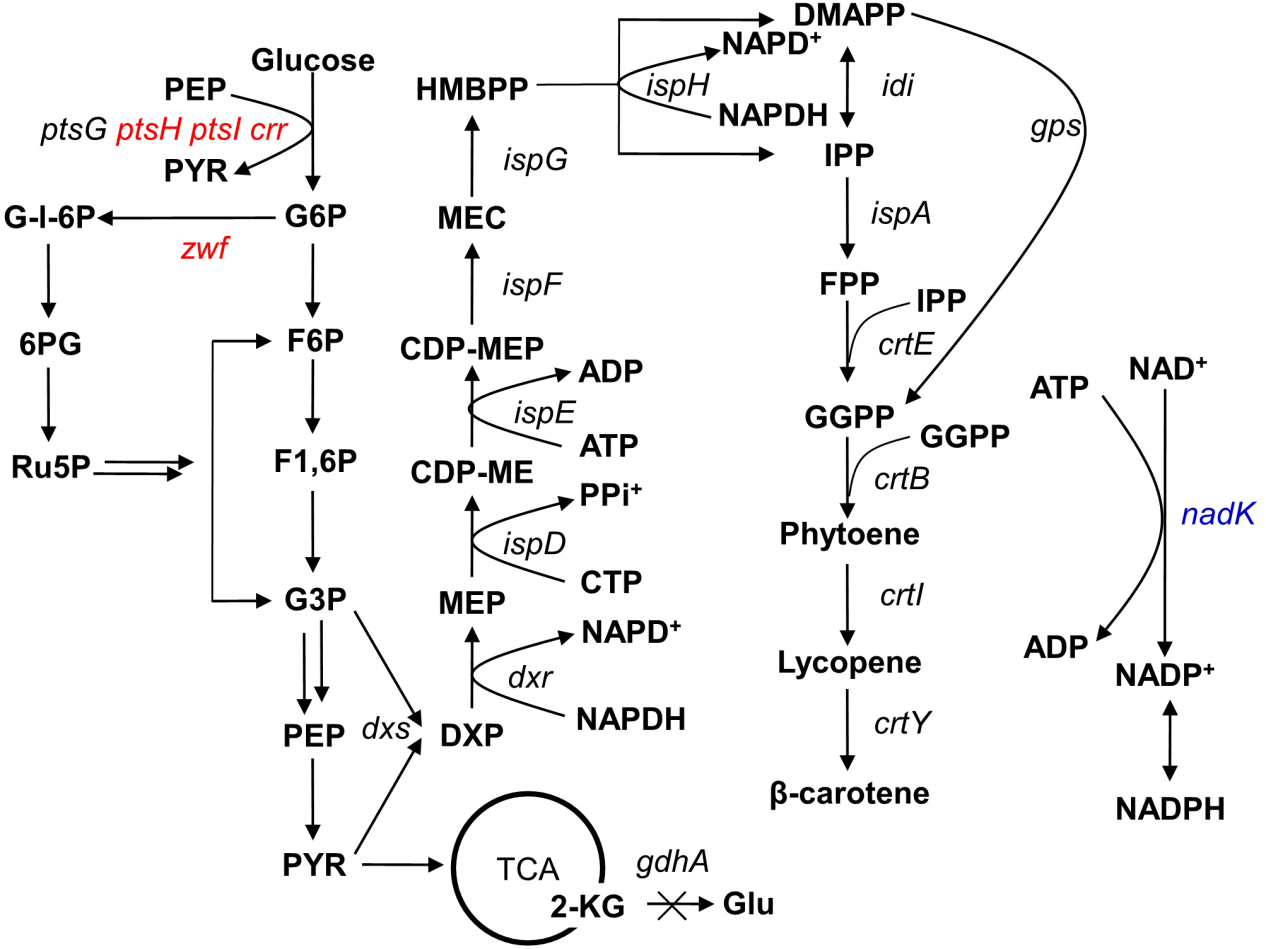
Simplified carbon metabolic profile of the producing *Escherichia coli* strain. The heterologous genes *gps* and *crtEBIY* were integrated into the *E. coli* genome at the *ldhA* locus to introduce the β-carotene pathway (Li et al., 2015). The genetic manipulations in this study are shown in colored fonts, where red represents gene deletion and blue represents gene overexpression. The multiple arrows represent multiple reactions. G6P, glucose-6-phosphate; F6P, fructose-6-phosphate; F1,6P, fructose-1,6-bisphosphate; G-l-6P, gluconolactone-6-phosphate; G3P, glyceraldehyde-3-phosphate; PEP, phosphoenolpyruvate; PYR, pyruvate; 6PG, 6-phosphogluconate; Ru5P, ribose-5-phosphate; 2-KG, 2-ketoglutaric acid; Glu, glutamic acid; DXP, 1-deoxy-d-xylulose-5-phosphate; MEP, 2C-methyl-d-erythritol-4-phosphate; CDP-ME, 4-diphosphocytidyl-2C-methyl-d-erythritol; CDP-MEP, 4-diphosphocytidyl-2C-methyl-d-erythritol-2-phosphate; MEC, 2C-methyl-d-erythritol-2,4-cyclodiphosphate; HMBPP, 1-hydroxy-2-methyl-2-(E)-butenyl-4-diphosphate; IPP, isopentenyl diphosphate; DMAPP, dimethylallyl diphosphate; GPP, geranyl diphosphate; FPP, farnesyl diphosphate; GGPP, geranylgeranyl diphosphate.

With the development of biotechnology, the fungus *Blakeslea trispora* and the microalga *Dunaliella salina* have been harnessed to produce natural β-carotene in large-scale fermentations (Aruldass et al., 2018). Moreover, metabolic engineering strategies have enabled the heterologous production of high levels of β-carotene by microbial cells (Wang et al., 2019). Many studies have focused on engineering *Escherichia coli* as an efficient heterologous producer of β-carotene. In *E*. *coli*, isoprenoid biosynthesis occurs through the methylerythritol phosphate (MEP) pathway, in which glyceraldehyde-3-phosphate and pyruvate are converted into two isoprenoid building blocks (viz., isopentenyl diphosphate (IPP) and dimethylallyl diphosphate) (Figure 1) with the help of cofactors cytidine triphosphate, adenosine triphosphate, and reduced nicotinamide adenine dinucleotide phosphate (NADPH). The overexpression of certain key enzymes in the MEP pathway, as well as strengthening of the precursor and cofactor supply, have proven to be effective strategies for increasing β-carotene production in *E*. *coli* (Sedkova et al., 2005; Yuan et al., 2006; Zhao et al., 2013; Li et al., 2015). In nature, mevalonate pathway, which exists in eukaryotes, archaea, and the cytosol of higher plants but not in *E*. *coli*, is another pathway for terpenoids biosynthesis. Acetyl-CoA is the origin of the pathway, and IPP is the end. Three molecules of acetyl-CoA are required for one molecule of IPP (Ye et al., 2016a). Some strategies that have been useful for increasing β-carotene production in *E*. *coli* include the introduction of the heterologous pathway to increase IPP supply, combinatorial expression of the mevalonate and MEP pathways, and optimization of the fermentation process (Yoon et al., 2009; Nam et al., 2013; Yang and Guo, 2014; Ye et al., 2016b). However, with regard to energy balance and product-glucose conversion, MEP pathway is more energetically balanced and exhibits higher (about 19.8%) mass yield of glucose for terpenoids production (Ye et al., 2016a).

A recent study identified 1-hydroxy-2-methyl-2-butenyl-4-diphosphate as a cytotoxic intermediate in the MEP pathway. The authors also achieved increased β-carotene production in *E*. *coli* by efficiently balancing the activation of two enzymes in the isoprenoid biosynthetic pathways, thereby eliminating the accumulation of the cytotoxic intermediates and allowing cell growth (Li et al., 2017). Subsequently, the same research group modulated the expression of membrane-bending proteins and engineered the membrane synthesis pathway to improve the cell's β-carotene storage capacity (Wu et al., 2017). They then constructed a novel artificial membrane vesicle transport system to excrete the hydrophobic β-carotene from the cells (Wu et al., 2019). These two strategies were applied in shake-flask cultures of the β-carotene-hyperproducing *E*. *coli* strain CAR025, where the total specific production of β-carotene was increased from 31.8 mg/g dry cell weight (DCW) to 44.2 mg/g DCW with the membrane engineering strategy, and from 27.2 to 44.8 mg/g DCW with the membrane vesicle trafficking system. In addition, since glycerol is the most favorable substrate for the production of isoprenoids in *E*. *coli* (Kim et al., 2010), one research group has constructed and optimized an alternative glycerol utilization pathway to improve β-carotene production (Guo et al., 2018).

In *E*. *coli*, phosphoglucose isomerase (*pgi*) and glucose-6-phosphate dehydrogenase (*zwf*) control the entry of carbon into Embden-Meyerhof-Parnas (EMP) pathway and pentose phosphate pathway (PPP), respectively. Glyceraldehyde-3-phosphate and pyruvate, the starting substrates of MEP pathway, are the key intermediate metabolites of the two pathways. It has been shown that 35–45% of NADPH is produced via PPP in *E*. *coli* (Sauer et al., 2004). It would seem that engineering carbon flux direct to the PPP is more beneficial for supplying the cofactors and balancing the precursors, and thus increasing carotenoids production via MEP pathway. However, through flux scanning based on enforced objective flux *in silico*, Choi et al. (2010) demonstrated that a decreased PPP flux and an increased glycolysis pathway flux could enhance lycopene production in *E*. *coli*. Subsequently, Zhou et al. (2013) confirmed that the deletion of *zwf* augmented the lycopene content in *E*. *coli*, whereas the deletion of *pgi* decreased the content.

Phosphoenolpyruvate (PEP) is the key nodes between glyceraldehyde-3-phosphate and pyruvate, and the enhanced supply increased lycopene production through overexpression of PEP synthetase (*ppsA*) or PEP carboxykinase (*pck*) in *E*. *coli* (Farmer and Liao, 2000, 2001). The phosphotransferase system (PTS) is the main glucose transport system in *E*. *coli*. When one molecule of glucose is transported via PTS, one molecule of PEP is consumed. This is unbeneficial for terpenoids production via MEP pathway. A previous study has shown that the increase in lycopene production by a PTS-inactivated *E*. *coli* strain was attributable to an increased phosphoenolpyruvate supply (Zhang et al., 2013). We have also previously demonstrated that deletion of the *ptsHIcrr* gene cluster in a *galP*-overexpressing strain resulted in a significant increase in β-carotene production (Li et al., 2015).

Previously, we had constructed a β-carotene-producing *E*. *coli* strain, ZF43Δ*gdhA*, which produced 116.2 mg/L of the provitamin with a content of 20.7 mg/g DCW (Li et al., 2015). This strain was characterized by the chromosomal overexpression of five heterologous genes (*crtE, crtB, crtI, crtY*, and *gps*, where *crtEBI* and *crtY*-*gps* are artificial operons in the *ldhA* locus) and five MEP pathway genes (*dxs, ispE, ispH, idi*, and *ispA*), and the deletion of the *gdhA* gene to decrease NADPH consumption (Figure 1). In this present study, we aimed to improve the β-carotene-producing capacity of strain ZF43Δ*gdhA* further by deleting its *zwf* and *ptsHIcrr* genes. The biomass and β-carotene production of the resultant strain ECW2 were significantly increased but β-carotene content was decreased. Subsequently, comparative transcriptomic analysis was used to explore the dynamic transcriptional responses of the strains to the blockade of PPP and inactivation of PTS. Finally, based on the analyses of comparative transcriptome and reduction cofactor, further strain manipulation was carried out to strengthen the NADPH supply, whereupon the β-carotene production by the best strain, ECW4/p5C-*nadK*, was 266.4 mg/L in flask culture and 2,579.1 mg/L in a 5-L bioreactor. The latter production value is the highest recorded via the MEP pathway in *E*. *coli*.

## Materials and Methods

### Strains, Plasmids, and Media

All strains and plasmids used in this study are listed in Table S1. During strain construction, cultures were grown aerobically (at 30°C and 220 rpm) in Luria-Bertani (LB) broth (10 g of tryptone, 5 g of yeast extract, and 10 g of sodium chloride, per liter) supplemented with appropriate antibiotics (10 mg/L kanamycin, 100 mg/L spectinomycin, and 10 mg/L tetracycline), 2 mM isopropyl-β-d-1-thiogalactopyranoside (IPTG), and 0.2% (*m*/*v*) l-arabinose. As the base medium for the other experimental processes, 2× YT medium (16 g of tryptone, 10 g of yeast extract, and 5 g of sodium chloride, per liter) supplemented with appropriate antibiotics was used.

### Genetic Manipulations

The strategies for marker-less gene deletion have been previously described in detail (Cox and Kuhlman, 2010; Lin et al., 2014). In brief, a linear DNA fragment consisting of the selectable marker *tetA* cassette flanked by homologous arms, duplication regions, and I-SceI recognition sites for targeted gene deletion was constructed through a single round of polymerase chain reaction (PCR). The pTKS/CS plasmid was used as the template, and each primer pair contained the sequences of the homologous arms, duplication regions, and I-SceI recognition sites (Table S2). The target DNA fragment was dispensed into 1-mm-gap cuvettes (Thermo Fisher Scientific, Waltham, MA, USA) containing pTKRED-carrying competent cells and electroporated into the cells at 1.8 kV, 25 μF, and 200 Ω using a gene pulser electroporation apparatus (Bio-Rad, Hercules, CA, USA). The tetracycline-resistant mutants were selected and confirmed by PCR. l-Arabinose and IPTG were subsequently added to induce I-SceI endonuclease expression for cleaving the *tetA* gene and to facilitate recombination between the duplication regions. The desired recombinant strains without the *tetA* gene were selected on l-arabinose plates and verified by PCR. *E*. *coli* strain DH5α was used as the clone host for recombinant plasmid construction. Plasmids p5C, p15C, and p20C were used as vectors for gene overexpression. To overexpress *pntAB, sthA, mdh*, and *nadK*, the DNA fragments of the four genes were amplified from the genome of *E*. *coli* K-12 MG1655, digested using the restriction enzymes *Sac*I and *Hin*dIII, and inserted into the corresponding sites of the respective vectors. For overexpression of the *tPOS5* gene, the 51 nt region of the 5′-terminus of the DNA fragment from the genome of *Saccharomyces cerevisiae* was truncated and amplified, and then digested and inserted into the vector in the same manner as for the four aforementioned genes. All recombinant plasmids were verified via sequencing by GENEWIZ, Inc. (Suzhou, China). The primers used are listed in Table S2.

### Physiological Analyses and β-Carotene Production in Shake Flask

The growth of the engineered strains and their glucose uptake were characterized. Cells of the strains from −80°C glycerol stocks were streaked onto LB agar plates and cultivated at 30°C. Single colonies were transferred from the plates into 30-mL glass tubes (15 × 150 mm) containing 3 mL of LB medium and cultured overnight at 30°C and 220 rpm. Subsequently, 50 mL of 2× YT medium containing 1% (*m*/*v*) glucose in a 250-mL flask was inoculated with 50 μL of the overnight culture and cultivated until the optical density at 600 nm (OD_600_) had reached ~0.8. The cultures were then inoculated at a final OD_600_ of 0.05 into 500-mL flasks containing 100 mL of 2× YT medium with 1% (*m*/*v*) glucose and incubated at 30°C and 220 rpm. Cultivation was carried out under aerobic conditions for 48 h. An appropriate volume of culture was sampled at every 2 h for determination of the OD_600_ and residual glucose concentration. The procedures of β-carotene production were the same as the physiological analysis, the difference was the sampling at 48 h for extraction. All media contained appropriate antibiotics (20 mg/L ampicillin and 10 mg/L kanamycin) and all experiments were performed in triplicate.

### Analytical Methods

For β-carotene quantification, intracellular β-carotene was extracted from the harvested cells through 15 min of acetone extraction at 55°C in the dark. Following centrifugation of the extract at 4°C and 10,000 rpm, the β-carotene content was quantified. All samples from shaking flask were quantified using high-performance liquid chromatography (HPLC, SHIMADZU, Kyoto, Japan) equipped with a HyPURITY C18 column (150 × 4.6 mm, 5 μm, Thermo Fisher Scientific, Inc., USA) at 470 nm. The mobile phase and gradient program had been previously described in detail (Zhou et al., 2015). The authentic β-carotene standard and retention time were as followed: β-carotene (≥ 95%, Sigma-Aldrich, USA), 22.8 min. During fed-batch fermentation, ultraviolet spectrophotometer (Beijing Puxi Universal, Co., Ltd, Beijing, China) was used to quantify the β-carotene production quickly as previously described (Li et al., 2015), and HPLC was used to re-quantify the production of some samples to confirm the quantitative accuracy of the absorption method after fermentation. Growth of the cells was monitored by measuring the OD_600_ with an ultraviolet spectrophotometer. For *E*. *coli*, the OD_600_ and DCW have the following conversion relation: 1 OD_600_ = 0.323 g DCW/L. Glucose in the culture broth was measured with an SBA-40C biosensor analyzer (Institute of Microbiology, Shandong, China). Acetate was determined by high-performance liquid chromatography on an apparatus (SHIMADZU, Kyoto, Japan) equipped with a refractive index detector and an Aminex HPX-87H column (300 × 7.8 mm; Bio-Rad). As the mobile phase, 5 mM H_2_SO_4_ was used at a flow rate of 0.6 mL/min at 65°C. The concentrations of intracellular NAD^+^, NADH, NADP^+^, and NADPH were measured according to the instructions provided in the quantitative kits (Beijing Solarbio Science and Technology Co., Ltd, Beijing, China), For the assay, 1 mL of each strain culture was sampled at 12 h and centrifuged at 4°C and 8,000 rpm for 10 min.

### Transcriptome Sequencing

Cell samples from each strain were prepared for transcriptome sequencing using the same method as used for the physiological analysis. At 12 h of culture, 10 mL of the cultured cells was harvested by centrifugation at 4°C and 8,000 rpm, washed three times using precooled phosphate-buffered saline (pH 7.0), frozen in liquid nitrogen, and sent to GENEWIZ Inc. for sequencing. Each strain was prepared in triplicate. The total RNA of each sample was extracted using the TRIzol reagent (Invitrogen, Carlsbad, CA, USA) and the RNeasy Mini Kit (Qiagen, Valencia, CA, USA), and quantified and qualified using a Bioanalyzer 2100 device (Agilent Technologies, Santa Clara, CA, USA) and NanoDrop spectrophotometer (Thermo Fisher Scientific) and by 1% (*m*/*v*) agarose gel electrophoresis. Paired-end sequencing libraries were prepared after depletion of rRNA, synthesis of cDNA, treatment of double-stranded cDNA ends, and amplification and purification using the corresponding kits. Libraries with different indices were multiplexed and loaded onto a HiSeq instrument according to the manufacturer's instructions (Illumina, San Diego, CA, USA). Sequencing was carried out using a 2 × 150 paired-end configuration. The sequencing data were deposited in the Sequence Read Archive database (SRA, https://www.ncbi.nlm.nih.gov/sra/) with accession number PRJNA607624. Image analysis and base calling were conducted with the HiSeq Control Software (HCS) + OLB + GAPipeline-1.6 (Illumina) algorithms on the HiSeq instrument. The sequences were processed and analyzed by GENEWIZ. Quality control of the sequencing reads was processed using Cutadapt v1.9.1 (Martin, 2011), and the high-quality reads were aligned to the *E*. *coli* K-12 MG1655 genome (GenBank Accession No. U00096.3) via Bowtie 2 (v2.2.5) (Langmead and Salzberg, 2012). The gene expression levels were estimated using HTSeq v0.6.1p1 with the reference genome, and differential expression analysis was performed using the DESeq2 Bioconductor package with the criteria of |log2 Fold Change| > 1.0 and *p*-value < 0.05. The ratio of transcripts per million was used to describe the relative expression strength of a gene in different strains (Wagner et al., 2012). Pathway enrichment analysis of the significantly differentially expressed genes was carried out against the Kyoto Encyclopedia of Genes and Genomes (KEGG) database, using in-house scripts. This analysis is based on KEGG pathway units and uses a hypergeometric test to determine the pathways of the differentially expressed genes that are significantly enriched against the transcriptome background. The formula used is as follows:

$$\begin{array}{l} p=1-\overset{m-1}{\underset{i=0}{\sum }}\frac{\left(\begin{array}{c} M\\ i \end{array}\right)\left(\begin{array}{c} N-M\\ n-i \end{array}\right)}{\left(\begin{array}{c} N\\ n \end{array}\right)} \end{array}$$

where *N* is the number of genes with pathway annotations; *n* is the number of differentially expressed genes in *N*; *M* is the number of genes annotated for a particular pathway in all genes; and *m* is the number of differentially expressed genes annotated for that pathway. The threshold used was *Q*-value ≤ 0.05.

### Fed-Batch Fermentation for β-Carotene Production

Fed-batch fermentation was carried out at 30°C in a 5-L bioreactor (B-type; T&J, Shanghai, China). The modified minimal medium used for seed preparation and fermentation contained (per liter) 10 g of glucose, 5 g of yeast extract, 1.7 g of citric acid, 24 g of KH_2_PO_4_, 4 g of (NH_4_)_2_HPO_4_, 1.01 g of MgSO_4_·7H_2_O, 4.5 mg of thiamine·HCl, and 1 mL of trace metal solution. The trace metal solution contained (per liter) 2.5 g of CoCl_2_·6H_2_O, 15 g of MnCl_2_·4H_2_O, 1.5 g of CuCl_2_·2H_2_O, 2.5 g of H_3_BO_3_, 2.5 g of Na_2_Mo_4_O_7_·2H_2_O, 2.5 g of Zn(CH_3_COO)_2_·2H_2_O, and 12.5 g of Fe(III)-citrate. Strain ECW4/p5C-*nadK* was first inoculated into 3 mL of 2 × YT medium for overnight culture. A portion of the culture was then transferred into 100 mL of modified minimal medium to create flask cultures at a starting OD_600_ of 0.05. The flask cultures were incubated at 30°C and 220 rpm for 24 h. The seed culture at an initial OD_600_ of 0.5 was then transferred to the bioreactor containing 2 L of fermentation medium. Fermentation was carried out at 30°C with an airflow rate of 1.5 vvm, and dissolved oxygen was kept at 30% by adjusting the agitation speed from 600 rpm to 1,000 rpm. The pH was maintained at 7.0 by the automatic addition of 5 M NH_3_·H_2_O. A solution composed of (per liter) 500 g of glucose, 2.5 g of yeast extract, and 7.5 g of MgSO_4_·7H_2_O was fed into the bioreactor at a feed rate of 7.5 mL/h.

## Results and Discussion

### Effect of *zwf* Gene Deletion on β-Carotene Production

**Figure 2:**
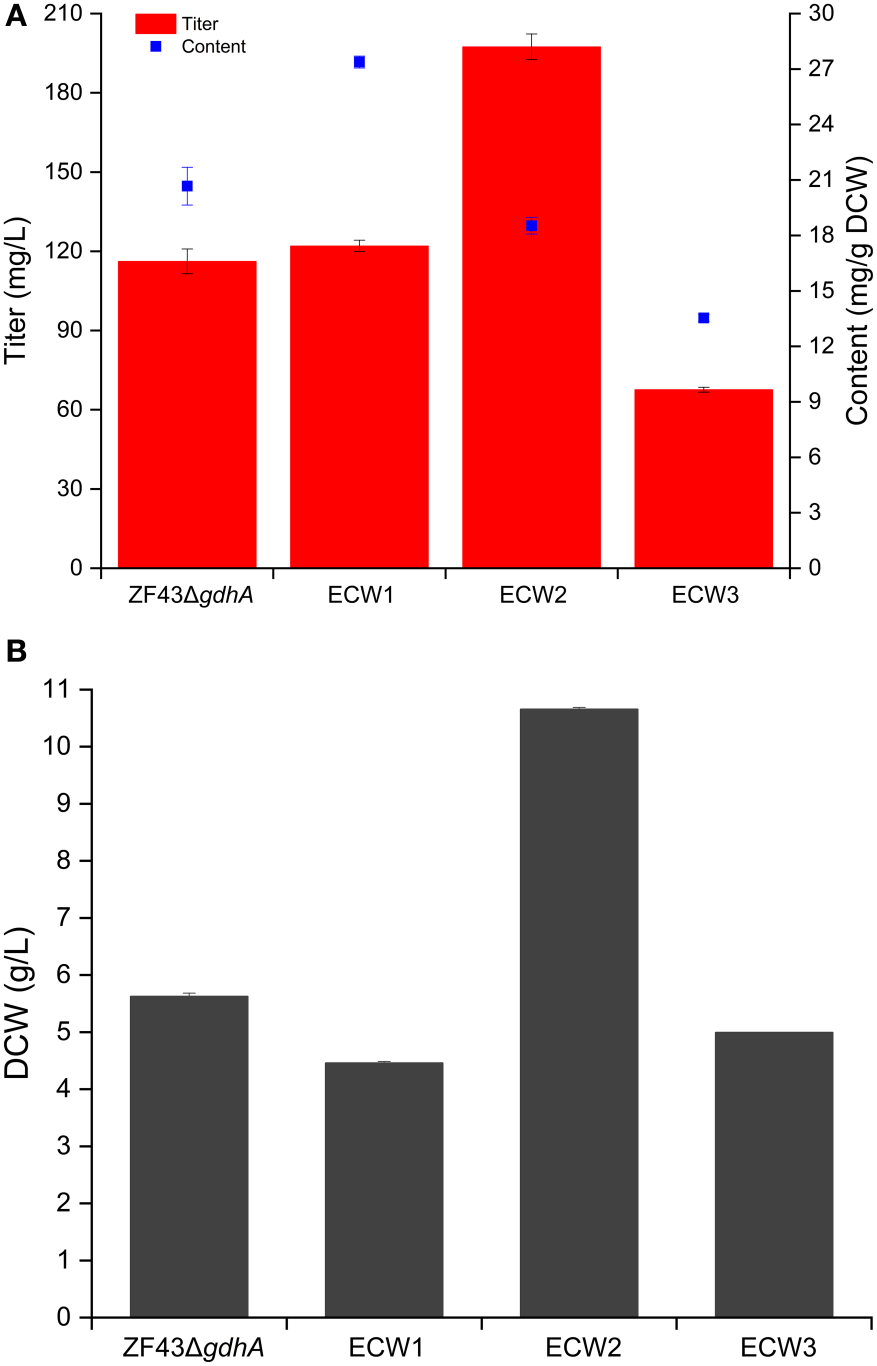
Effects of *zwf* deletion and phosphotransferase system inactivation on β-carotene production **(A)** and biomass generation **(B)** in *Escherichia coli* strain ZF43Δ*gdhA*. The results represent the means from four independent experiments, and the error bars represent standard deviations.

Zhou et al. (2013) validated that *zwf* deletion rather than *pgi* deletion was beneficial for the lycopene content in *E*. *coli*. Considering that lycopene is the direct precursor of β-carotene in *E*. *coli*, we deleted the *zwf* gene in *E*. *coli* strain ZF43Δ*gdhA* to explore the effect of its knockout on β-carotene production in the resultant strain ECW1 (ZF43Δ*gdhA*Δ*zwf*). Compared with that by strain ZF43Δ*gdhA*, β-carotene production by strain ECW1 increased by 5.1% only (from 116.2 to 122.0 mg/L) (Figure 2A). However, the β-carotene content increased by 32.5% (from 20.7 to 27.4 mg/g DCW), which was due to the much lower (26.2%) biomass of the strain (Figure 2B). Previous studies had indicated that the strength of *zwf* transcription was decreased in lycopene-overproducing strains of *E*. *coli* (Mickus, 2009), and that deletion of the gene decreased the biomass but increased lycopene content (Zhou et al., 2013). Our results indicated that the effect of *zwf* deletion on β-carotene production was similar to its effect on lycopene production.

We tested the maximum specific growth rates and specific glucose uptake rates of the strains (Table 1). Strain ZF43Δ*gdhA* took ~24 h to fully consume 10 g/L glucose, while strain ECW1 took ~26 h (Figure S1). Strain ECW1 had a lower maximum specific growth rate, but a higher specific uptake rate, than strain ZF43Δ*gdhA*. These results are consistent with previous reports that *zwf* deletion decreased the cell growth rate but increased the substrate uptake rate during the growth of *E. coli* on glucose (Zhao et al., 2004a; Zhou et al., 2013).

**Table 1:** Maximum specific growth rates and average specific glucose uptake rates of *Escherichia coli* strains ZF43Δ*gdhA*, ECW1, ECW2, and ECW3.

**Strains**	**Maximum specific growth rate (h^**−1**^)**	**Specific glucose uptake rate^a^ (mmol g DCW^**−1**^ h^**−1**^)**
ZF43Δ*gdhA*	0.693 ± 0.015	1.032 ± 0.018^b^
ECW1	0.656 ± 0.002	1.168 ± 0.013^b^
ECW2	0.565 ± 0.001	0.673 ± 0.033^c^
ECW3	0.535 ± 0.002	0.295 ± 0.001^b^

a *The specific glucose uptake rates were calculated during the transition phase between log-growth and stational phases of three strains. The specific glucose uptake rate of ECW2 was calculated at a different time due to its much longer log-growth phase and glucose consumption time (Figure S1). It was notable that components in the 2 × YT medium also contributed to the biomass yield*.

b *Calculated on the basis of 8–18 h*.

c *Calculated on the basis of 20–30 h*.

*The results represent the means from three independent experiments*.

For biosynthesis of one β-carotene molecule via MEP pathway, eight adenosine triphosphate, eight cytidine triphosphate, and 16 NADPH molecules are required. PPP pathway is the main pathway to supply NADPH in *E*. *coli* (Sauer et al., 2004). However, previous studies have shown that overexpression of key PPP enzymes did not increase the production of β-carotene significantly (Zhao et al., 2013), and the β-carotene-hyperproducing *E*. *coli* strains (e.g., recombinant DH5α, CAR005, and ZF237T) did not contain any PPP modifications (Nam et al., 2013; Zhao et al., 2013; Li et al., 2015). In addition, we also deleted the *pgi* gene in strain ZF43Δ*gdhA*, whereupon the resultant strain only produced 5.1 mg/L of β-carotene. The metabolic flux analysis indicated that the *zwf* gene deletion had increased the carbon flux through the tricarboxylic acid (TCA) cycle to generate more reducing equivalents and supplement the shortage, while simultaneously increasing the glycolytic flux significantly (Zhao et al., 2004b). This might have increased the supply of glyceraldehyde-3-phosphate and pyruvate and thus improved β-carotene production.

### Effect of Phosphotransferase System Inactivation on β-Carotene Production in Strain ECW1

Previous studies have shown that the increase in carotenoids productions by PTS-inactivated *E*. *coli* strains were attributable to an increased PEP supply (Zhang et al., 2013; Li et al., 2015). Thus, in this study, the *ptsHIcrr* gene cluster was deleted in strains ECW1 and ZF43Δ*gdhA* to further increase β-carotene production, resulting in strains ECW2 and ECW3, respectively. Unexpectedly, strain ECW2 produced 10.7 g/L of biomass and 197.4 mg/L of β-carotene, which represented increases of 139.1 and 61.8% over those of strain ECW1, respectively. By contrast, strain ECW3 only produced 5.0 g/L of biomass and 67.6 mg/L of β-carotene, which were, respectively, 11.2 and 41.2% lower than those of strain ZF43Δ*gdh* (Figure 2A). The β-carotene contents of the two strains were lower than those of their respective parental strain. However, strain ECW2 exhibited a much longer log-growth phase and could fully consume 10 g/L of glucose in ~36 h (Figure S1); thus, its glucose uptake was significantly decreased (Table 1). Although strain ECW3 also exhibited significantly decreased glucose uptake (Table 1), it could not fully consume 10 g/L of glucose within 48 h (Figure S1).

We could not detect acetate in the culture broth of strain ECW3, which was similar to that observed in the PTS-inactivated *E*. *coli* strain PB11 (Flores et al., 2002). This may be related to the internal transformation of acetate to acetyl-CoA by acetyl-CoA synthetase within the cell (Flores et al., 2005). By contrast, acetate was detected in the culture broths of strains ZF43Δ*gdhA*, ECW1, and ECW2 when the glucose was nearly exhausted during their fermentation. Strains ZF43Δ*gdhA* and ECW1 produced 1.2 and 2.9 g/L of acetate, respectively, when glucose was exhausted, whereas strain ECW2 only produced 0.27 g/L of acetate by 36 h of culture. The deletion of *zwf* increased acetate accumulation in strain ECW1, which further led to the lower biomass of the strain relative to that of its parental strain. Zhao et al. (2004a) also demonstrated an increase in the acetate production rate to 1.11 mmol g^−1^ h^−1^ in the *zwf*-knockout *E*. *coli* strain BW25113 relative to the 0.58 mmol g^−1^ h^−1^ rate in the wild-type strain (Zhao et al., 2004a). The increased overflow metabolism in the *zwf*-knockout strain was due to the higher average specific glucose uptake rate (Table 1), which resulted from the much higher activities of the key enzymes (phosphoglucose isomerase and isocitrate dehydrogenase) located in the EMP pathway and TCA cycle (Zhao et al., 2004a). However, inactivation of the PTS significantly decreased the overflow metabolism in strain ECW2, which was beneficial for biomass accumulation, and increased β-carotene production in *E*. *coli* (Luli and Strohl, 1990; Eiteman and Altman, 2006).

It has been shown that PTS inactivation together with strain evolution could increase the production of aromatic compounds and succinate in *E*. *coli* as a result of the increased phosphoenolpyruvate concentration caused by the PTS inactivation (Floras et al., 1996; Gosset, 2005; Thakker et al., 2012). However, the glucose utilization rates and biomasses of the PTS-inactivated *E*. *coli* strains were significantly decreased (Floras et al., 1996; Wang et al., 2006; Lu et al., 2012). With regard to lycopene production, the biomass of *E*. *coli* strain PTS01 was significantly improved through medium optimization, but did not exceed that of wild-type strain MG01 after isopropyl β-thiogalactopyranoside induction (Zhang et al., 2013). In this present study, the glucose utilization rate of strain ECW2 was 60.7% of that of strain ECW1 (Table 1) and the biomass was significantly increased (Figure 2B). As for β-carotene production in *E*. *coli*, the biomass and β-carotene production were decreased significantly when *galP* was over-expressed, then, the biomass was restored and production was increased significantly after *ptsHIcrr* deletion (Li et al., 2015). Here, *zwf* deletion increased β-carotene production and decreased biomass, subsequently, *ptsHIcrr* deletion significantly enhanced the production and biomass of strain ECW2. The double deletion coupled the biomass and β-carotene production of strain, and was efficient for improving production. To our best knowledge, this is a novel combination for β-carotene production in *E*. *coli*, and will be useful for the production of other carotenoids. System biology analyses should be carried out to explore the responses of cells to the double deletions.

### Comparative Transcriptomic Analysis of Strains ZF43Δ*gdhA*, ECW1, and ECW2

To explore the transcriptional responses of the *E*. *coli* strains to the *zwf* and *ptsHIcrr* deletions, strains ZF43Δ*gdhA*, ECW1, and ECW2 were transcriptionally profiled. Cell proliferation experiments indicated that these three strains were in the exponential growth phase and had differences in biomass after 12 h of culture (Figure S1). The gene expression levels in the 12-h cultures of the three strains were assessed, and comparative transcriptomic analysis was carried out.

**Figure 3:**
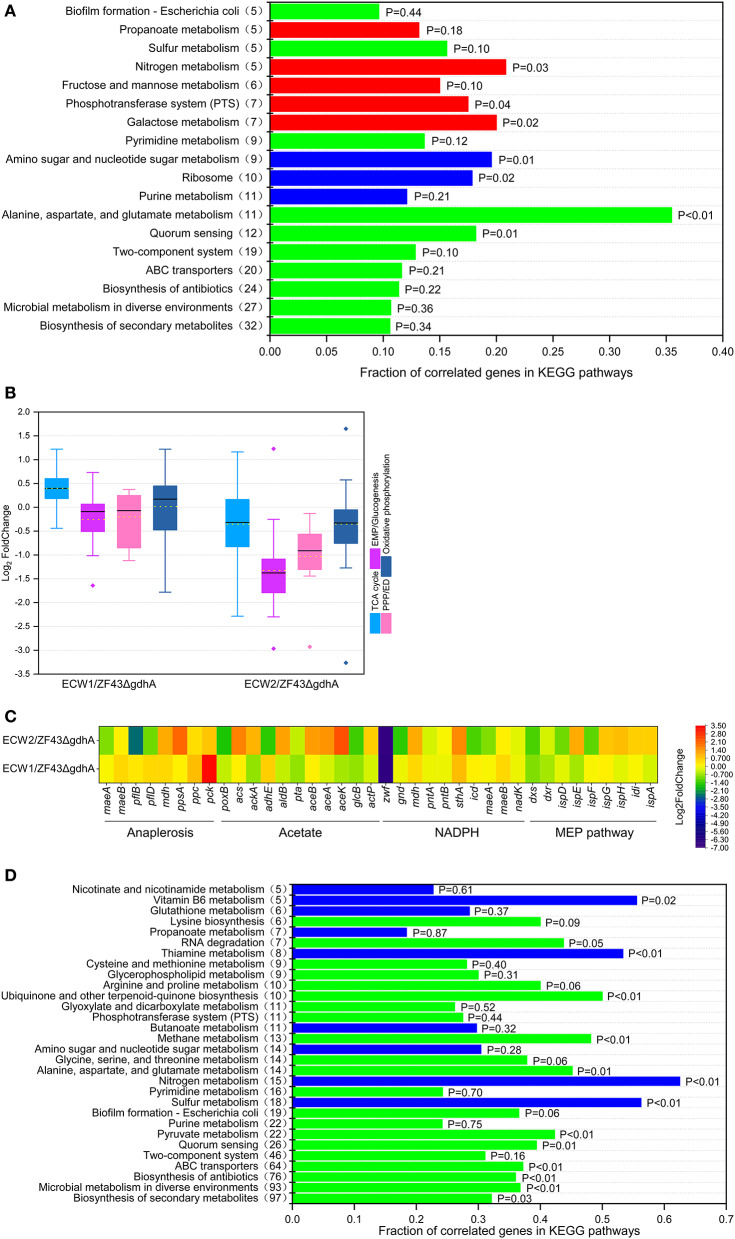
Comparative transcriptomic analysis of *Escherichia coli* strains ZF43Δ*gdhA*, ECW1, and ECW2. **(A)** Fraction of correlated genes in KEGG pathways of strain ECW1 relative to those of strain ZF43Δ*gdhA*. The *x*-axis indicates the ratio of the number of genes differentially expressed in the pathway relative to the total number of genes in the pathway. The *y*-axis numbers in parentheses are the number of genes differentially expressed in the pathway. Red indicates an upregulated pathway, blue a downregulated pathway, and green a pathway containing both upregulated and downregulated genes. **(B)** Box plot of the log_2_FoldChange of the differentially expressed genes participating in the TCA cycle, EMP/gluconeogenesis pathway, pentose phosphate pathway, and oxidative phosphorylation in strains ECW1 and ECW2 relative to those in strain ZF43Δ*gdhA*. The bold line in the box indicates the median and the yellow dot indicates the mean. The lower and upper bounds of the box indicate the first and third quartiles, respectively, and the whiskers show ±1.5× the interquartile range. **(C)** Transcriptional levels of genes related to the anaplerotic pathways, acetate pathways, NADPH pathways, and methylerythritol phosphate (MEP) pathways in strains ECW1 and ECW2 relative to those in strain ZF43Δ*gdhA*. **(D)** Fraction of correlated genes in KEGG pathways of strain ECW2 relative to those of strain ECW1. The meaning of the colors is the same as in **(A)**.

Among the 4,583 predicted genes of *E*. *coli* K-12, 452 genes (including *zwf*) were differentially expressed between strains ECW1 and ZF43Δ*gdhA*. These genes participated in nearly all biological processes, such as “biosynthesis of secondary metabolites,” “ABC transporters,” “two-component systems,” and “alanine, aspartate, and glutamate metabolism” (Figure 3A). Further, the expression levels of genes participating in the *de novo* synthesis of purine and formation of the large subunit of ribosome were significantly decreased (Figure S2). The reduced expression of these genes should have contributed to the lower maximum specific growth rate and biomass of strain ECW1. In central carbon metabolism, the expression level of the EMP pathway and PPP genes were predominantly downregulated, and the TCA cycle genes were predominantly upregulated in strain ECW1 (Figure 3B). Moreover, the expression levels of *aceE* and *aceF* were downregulated in this strain, whereas those of the anaplerotic pathway genes *ppc, pflB*, and *pflD* were upregulated (Figure 3C). Furthermore, propanoate metabolism pathway linking acetyl-CoA to succinate was also upregulated (Figure 3A). We inferred that the upregulated anaplerotic pathways supplied adequate acetyl-CoA and oxaloacetate for the strengthened TCA cycle. The imbalance in transcriptional profile of central carbon metabolisms in strain ECW1 might be one of reasons for the increased overflow metabolism. Nevertheless, a previous study had shown that strengthening TCA cycle resulted in increases in the energy and reduction power to increase β-carotene production (Zhao et al., 2013). *pck* expression was significantly upregulated in strain ECW1, being 3.3-fold higher than that in strain ZF43Δ*gdhA* (Figure 3C). It was previously demonstrated that *pck* overexpression was beneficial for lycopene production in *E*. *coli* owing to the increased PEP supply (Farmer and Liao, 2001). For NADPH generation, the expression of *pntA* and *pntB* was not affected, but that of *mdh, mqo, maeB*, and *sthA* was increased (Figure 3C and Table S3). In the MEP pathway, the expression strengths of *dxs, dxr*, and *ispE* in strain ECW1 were 54, 64, and 62% of those in strain ZF43Δ*gdhA*, respectively (Table S3). In strain ZF43Δ*gdhA, dxs* is controlled by the strong constitutive promoter J23119 (Li et al., 2015), and its transcripts per million value was very high (1.89). Although the strength of *dxs* expression was decreased by 46% in strain ECW1 relative to that in strain ZF43Δ*gdhA*, the value (transcripts per million value of 1.02) was still higher than that of other genes in the MEP pathway (Table S3). Hence, the carbon flux toward IPP and dimethylallyl diphosphate biosynthesis was efficient. Moreover, the expression strengths of the heterologous genes *crtE, crtB, crtI, crtY*, and *gps* of strain ECW1 were 115, 108, 113, 91, and 118% of those of strain ZF43Δ*gdhA*, respectively. These results might have contributed to the increased β-carotene content in strain ECW1.

Unlike those of strain ECW1, the exponential growth phase, final biomass, and β-carotene production of strain ECW2 were significantly increased (Figure 2, Figure S1). Transcriptomic analysis of strain ECW2 showed that the expression of 1,116 genes was significantly changed relative to their expression in strain ZF43Δ*gdhA*, with 541 being notably upregulated and 575 (including genes *zwf*, *ptsH, ptsI*, and *crr*) being downregulated. Compared with the levels in strain ZF43Δ*gdhA*, the expression levels of genes involved in central carbon metabolism (“glycolysis/gluconeogenesis,” the “TCA cycle,” and the “pentose phosphate pathway”), energy metabolism (“oxidative phosphorylation,” “sulfur metabolism,” and “nitrogen metabolism”), and cofactor metabolism (“vitamin B6 metabolism” and “nicotinate and nicotinamide metabolism”) were significantly downregulated in strain ECW2 (Figures 3B,D). In addition, *aceE, aceF, lpd, pflB, pflD*, and propanoate metabolism pathway were significantly downregulated (Figures 3B–D). These were consistent with the downregulated central carbon metabolism. The depressed central carbon metabolism and energy metabolism pathways of this strain may be related to its decreased glucose uptake rate. In addition, the expression levels of genes *poxB* and *adhE* (both involved in acetate formation) in strain ECW2 were significantly lower, whereas those of genes *acs, ackA, aceA, aceB*, and *aceK* (all involved in acetate utilization) were 1.68-, 0.97-, 1.45-, 1.37-, and 2.35-fold higher, than those of the corresponding genes in strain ZF43Δ*gdhA* (Figure 3C). These results could explain the reason that strain ECW2 produced small titer of acetate. We inferred that the different expression levels of these genes had positive effects on biomass generation. The expression of genes involved in NADPH generation was decreased, except for that of *mdh* and *sthA* (Figure 3C). In the MEP pathway, the expression levels of *ispE, ispG, ispH, idi*, and *ispA* were 166, 128, 135, 119, and 118% of those of strain ZF43Δ*gdhA*, respectively. However, the expression levels of the key genes *dxs, ispD*, and *ispF* were downregulated significantly in strain ECW2 (Table S3). As for the β-carotene biosynthetic genes in strain ECW2, the expression levels of *crtE, crtB*, and *crtI* were significantly decreased, whereas the expression of *crtY* was unchanged, which may have contributed to the decreased β-carotene content (Table S3). PTS inactivation in strain ECW1 depressed the central carbon and energy metabolism pathways, but might achieve a new balance for strain ECW2 in global transcriptional profile, and this allowed for better coordination of the carbon flux distribution (Chubukov et al., 2014), coupled with the upregulated acetate utilization pathways. Hence, strain ECW2 outperformed ECW1 in terms of biomass. By contrast, PTS inactivation in strain ECW1 depressed NADPH generation and the MEP and β-carotene biosynthetic pathways, resulting in the decreased intracellular β-carotene content in strain ECW2.

### Analysis of Reduction Cofactor in Strains ECW1 and ECW2

In the oxidative PPP, oxidation of glucose-6-phosphate catalyzed by glucose-6-phosphate dehydrogenase and decarboxylation of 6-phosphogluconate catalyzed by 6-phosphogluconate dehydrogenase both produce NADPH. Given that NADPH is an indispensable cofactor for β-carotene biosynthesis in *E*. *coli* and that the deletion of *zwf* definitely reduced the supply of this cofactor through the PPP. Conversely, comparative transcriptomic analysis indicated that the expression levels of genes involving NADPH generation and NADH were increased in strain ECW1, whereas the levels were decreased in strain ECW2. To ascertain whether the strain engineering had improved the cofactor supply, we measured the levels of NADH and NADPH in strains ZF43Δ*gdhA*, ECW1, and ECW2 (Table 2). In strain ECW1, the level of NADH was increased by 70.5% relative to that in strain ZF43Δ*gdhA*, and the total level of coenzyme I was increased by 9.8%. These results were consistent with those of a previous report, in that *zwf* deletion produced higher fluxes through the TCA cycle and then generated more NADH (Zhao et al., 2004a). In the case of coenzyme II, the NADPH level in strain ECW1 was decreased by 12.5% compared with that in strain ZF43Δ*gdhA*, as expected, but the total level of coenzyme II was slightly decreased (1.3%) as well. The NADPH/NADP^+^ ratio in strain ECW1 was decreased by 21.1%, but the NADH/NAD^+^ ratio was increased by 81.8%, relative to the ratios in strain ZF43Δ*gdhA*. The enhanced fluxes through the TCA cycle could quickly supplement the NADPH required for higher β-carotene synthesis through the transformation of NADH to NADPH (Zhao et al., 2004b). The higher NADH/NAD^+^ ratio was advantageous for this transformation, and might be one of the main factors for the enhanced β-carotene content in strain ECW1 (Figure 2). However, the low NADPH/NADP^+^ ratio in strain ECW1 suggested that its NADPH supply could be a limiting factor to improving β-carotene production.

**Table 2:** Specific intracellular concentrations (μmol/g DCW) of NADH and NADPH in *Escherichia coli* strains ZF43Δ*gdhA*, ECW1, and ECW2 at 12 h of fermentation.

**Strains**	**NADH**	**NAD^**+**^**	**NADH+NAD^**+**^**	**NADH/NAD^**+**^**	**NADPH**	**NADP^**+**^**	**NADPH+NADP^**+**^**	**NADPH/NADP^**+**^**
ZF43Δ*gdhA*	0.25 ± 0.01	0.96 ± 0.05	1.21 ± 0.04	0.27	3.83 ± 0.04	3.52 ± 0.05	7.35 ± 0.01	1.09
ECW1	0.43 ± 0.00	0.90 ± 0.03	1.33 ± 0.02	0.48	3.36 ± 0.07	3.90 ± 0.03	7.25 ± 0.09	0.86
ECW2	0.21 ± 0.02	1.14 ± 0.04	1.35 ± 0.06	0.18	3.20 ± 0.09	3.86 ± 0.03	7.06 ± 0.06	0.83

*The results represent the means from three independent experiments*.

For the PTS-inactivated strain ECW2, the NADH level was decreased by 51.2%, whereas the NAD^+^ level was increased by 26.7%, relative to the levels in ECW1. Thus, its NADH/NAD^+^ ratio was 62.5% lower than that of strain ECW1 (Table 2), which was unfavorable for NADH-to-NADPH conversion and could lead to a decrease in the NADPH level. Unexpectedly, the NADPH level in strain ECW2 was only slightly lower than that in strain ECW1, which might be due to a lower NADPH consumption rate resulting from the much lower growth rate of strain ECW2. The β-carotene content in strain ECW2 was 32.3% lower than that in strain ECW1 (Figure 2A). Thus, we speculated that the NADPH required for β-carotene biosynthesis could be insufficient in strain ECW2 owing to the decreased conversion rate from NADH to NADPH. Hence, increasing the NADPH supply was the focus of the following work.

### Increasing the NADPH Supply to Increase β-Carotene Production in Strain ECW2

**Figure 4:**
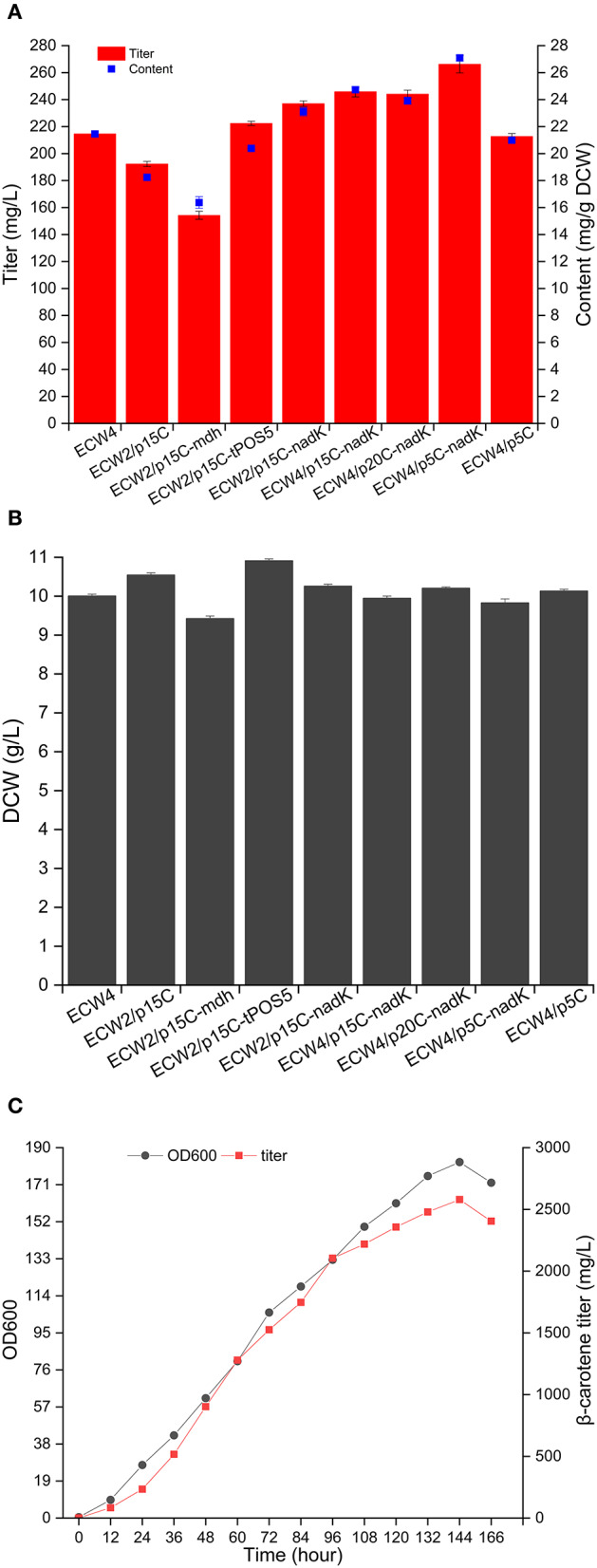
NADPH supply to increase β-carotene production by different engineered *Escherichia coli* strains. Production of β-carotene **(A)** and biomass **(B)** by the different strains. The results represent the means from four independent experiments, and the error bars represent standard deviations. **(C)** Fed-batch fermentation of strain ECW4/p5C-*nadK*. Ultraviolet spectrophotometer was used to quantify the β-carotene production quickly during the fed-batch fermentation, and HPLC was used to re-quantify the production of some samples to confirm the quantitative accuracy of the absorption method after fermentation. Results showed that the quantifications obtained from the two methods were consistent.

Generally, there are three strategies that can be used to increase the NADPH supply in *E*. *coli*: reducing NADPH consumption, strengthening NADPH generation, and converting NADH to NADPH (Zhou et al., 2017). Previous studies have shown that deletion of *yjgB*, which encodes a strong NADPH-dependent aldehyde reductase, could mediate the increase in geraniol and protoilludene production in *E*. *coli* (Zhou et al., 2014, 2017). Therefore, the *yjgB* gene was deleted in strain ECW2, yielding strain ECW4. The β-carotene production amount and content in strain ECW4 were 214.6 mg/L and 21.5 mg/g DCW, respectively, which were 8.7 and 15.8% higher than those in strain ECW2, whereas the biomass of strain ECW4 was only 6.1% less than that of strain ECW2 (Figures 4A,B). In addition, many studies have shown that overexpression of the native genes *mdh, pntAB, sthA*, and *nadK* (*yfjB*) and the heterologous gene *tPOS5p* from *S*. *cerevisiae* could increase the production of targeted chemicals (including terpenoids) in *E*. *coli* due to the increased NADPH supply (Sánchez et al., 2006; Li et al., 2009; Choi et al., 2010; Lee et al., 2013; Shi et al., 2013; Zhao et al., 2013; Zhou et al., 2017). Therefore, in this study, the *mdh, pntAB, sthA, nadK*, and *tPOS5p* genes were separately inserted into plasmid p15C for their overexpression using the constitutive promoter apFAB72 and the ribosome-binding site (RBS) apFAB848, which have moderate transcription and translation strengths, respectively (Kosuri et al., 2013). We could not transform strain ECW2 with the recombinant plasmids p15C-*pntAB* and p15C-*sthA*. We speculate that overexpression of genes *pntAB* and *sthA* may have destroyed the chemical equilibrium between the pyridine pools (Lee et al., 2013) and was lethal for the host strain. β-Carotene production by strain ECW2 harboring plasmid p15C-*mdh* was lower than that by strain ECW2/p15C. By contrast, strains ECW2/p15C-*nadK* and ECW2/p15C-*tPOS5p* produced 237.0 mg/L (23.1 mg/g DCW) and 222.4 mg/L (20.4 mg/g DCW) of β-carotene, respectively (Figure 4A), which were 23.2% (26.7%) and 15.6% (11.8%) higher than the level in strain ECW2/p15C.

Subsequently, the deletion of *yjgB* and the use of the *nadK* expression cassette to overexpress NAD kinase were combined to further increase β-carotene production. This combination strategy was based on the lower β-carotene content produced by strain ECW2/p15C-*tPOS5p* (Figure 4A) and the genetic heterogeneity of heterogeneous expression (Rugbjerg and Sommer, 2019). Consequently, the β-carotene production amount and content of ECW4/p15C-*nadK* were further increased to 245.9 mg/L and 24.7 mg/g DCW, respectively.

To test the effects of plasmid copy number on β-carotene production, the *nadK* expression cassette was transferred to the low-copy-number plasmid p5C and high-copy-number plasmid p20C. The two resulting plasmids were then used to transform strain ECW4. Interestingly, the highest β-carotene production amount and content (266.4 mg/L and 27.1 mg/g DCW, respectively) were achieved by strain ECW4/p5C-*nadK*, representing increases of 25.2 and 29.1%, respectively, over those of strain ECW4/p5C (Figure 4A). The production titer was comparable to the 268.1 mg/L produced by strain CAR025-37Almgs(pPlsb-plsc) in LB medium containing 20 g/L of glycerol (Wu et al., 2017). However, we failed to increase β-carotene production by modulating the expression level of *nadK* on the genome using three RBSs with different strengths (Figure S3). The construction of a plasmid-free strain capable of high β-carotene production is worthy of future study. Finally, strain ECW4/p5C-*nadK* was fed-batch cultured in a 5-L bioreactor. The maximum OD_600_ reached 182, and the highest β-carotene production amount reached 2,579.11 mg/L at 144 h (Figure 4C). To the best of our knowledge, this production titer is the highest reported for an *E*. *coli* strain via an engineered MEP pathway. Certainly, the time of fermentation is slightly long for engineered *E*. *coli*. The most important reason is that the central carbon metabolism was repressed caused by the double deletion of *zwf* and *ptsHIcrr*. The second reason was that the fermentation process was not optimal. In the future, fermentation efficiency should be improved through genetic modification of potential targets and optimization of fermentation process such as medium and fed-batch optimization (Lee, 1996).

## Conclusions

In this study, we successfully enhanced β-carotene production in *E*. *coli* by perturbing central carbon metabolism and improving the NADPH supply. Blocking the PPP and inactivating the PTS increased the β-carotene production capacity of strain ECW2 by 70.0% over that of the parental strain ZF43Δ*gdhA*. Although the β-carotene content of strain ECW2 was lower than that of strain ECW1, regulating the NADPH supply in strain ECW2 restored the content and further increased the production of the provitamin, with strain ECW4/p5C-*nadK* showing the highest production titer of 266.24 mg/L, an increase of 34.9% over that of the parental strain ECW2. This study, to the best of our knowledge, is the first to prove the efficient combination of double deletions of *zwf* and *ptsHIcrr* for β-carotene production in *E*. *coli*, although the strategy applied is quite routine. Comparative transcriptomic analyses indicated that the double deletions depressed the central carbon and energy metabolism pathways, but upregulated the acetate utilization pathway. These might have better coordinated the carbon flux distribution within cells, which was beneficial for biomass and β-carotene generation.

Although the new combination is efficient for β-carotene production, some key enzymes in the MEP and β-carotene biosynthetic pathways were downregulated, especially the one encoded by gene *ispD*. It is highly possible that the production of β-carotene in strain ECW4/p5C-*nadK* can be further increased via the optimization of these two pathways, using strategies such as promoter replacement, copy number increase, and RBS regulation. In addition, the fermentation efficiency of this strain was unsatisfactory in this work, as the fermentation time was slightly long for *E*. *coli*. Therefore, fermentation optimization will be indispensable for increasing the efficiency of the strain. Lastly, it is undoubtable that the genetic stability of strain ECW4/p5C-*nadK* is not robust owing to the recombinant plasmid. Although we failed to improve β-carotene production via the RBS replacement of *nadK* in the genome, increasing the copy number of this gene in the genome should be useful in the future. Furthermore, considering that balanced co-expression of MEP and mevalonate pathways effectively increased β-carotene production in *E*. *coli* (Yang and Guo, 2014; Ye et al., 2016b), mevalonate pathway will be introduced into our strain to increase β-carotene production, and combinatorial regulation of the two pathways should be carried out.

In conclusion, the new combinations reported for the first time in this study—involving only the deletion of two genomic sites and modification of the NADPH supply—are simple yet efficient and may help to produce many other natural products, especially isoprenoids. More importantly, we demonstrated that the use of the MEP pathway alone for efficient β-carotene biosynthesis could be achieved via appropriate modifications of the cell metabolic functions.

## Data Availability Statement

The sequencing data were deposited in the Sequence Read Archive database (SRA, https://www.ncbi.nlm.nih.gov/sra/) with accession number PRJNA607624.

## Author Contributions

TC and YW designed the study and wrote the manuscript. YW, PY, YL, XL, and ZW performed the experiments and analyzed the results. XZ and TC supervised the project and critically revised the manuscript. All authors gave final approval of the version to be submitted, and read and approved the final manuscript.

## Conflict of Interest

The authors declare that the research was conducted in the absence of any commercial or financial relationships that could be construed as a potential conflict of interest.

## Abbreviations

MEP: methylerythritol phosphate
IPP: isopentenyl diphosphate
PPP: pentose phosphate pathway
EMP: Embden-Meyerhof-Parnas
PTS: phosphotransferase system
PEP: phosphoenolpyruvate
DCW: dry cell weight
TCA: tricarboxylic acid.
