# Supplementary material for: Enhancing β-Carotene Production in Escherichia coli by Perturbing Central Carbon Metabolism and Improving the NADPH Supply
Source: Front Bioeng Biotechnol. 2020 Jun 9;8:585. doi: 10.3389/fbioe.2020.00585 (PMC7296177; doi:10.3389/fbioe.2020.00585)
Supplement: Supplementary file 1 [file Data_Sheet_1.pdf]

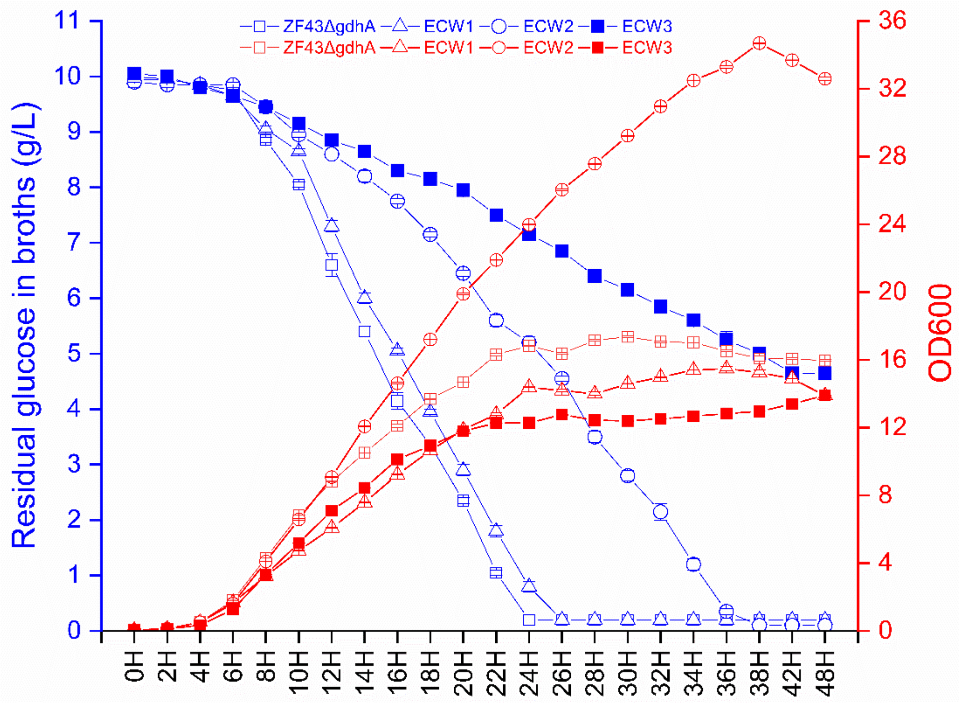


**Supplementary Figure 1.** Growth (red lines) and residual glucose (blue lines) curves for *Escherichia coli* strains ZF43Δ*gdhA*, ECW1 and ECW2.

A

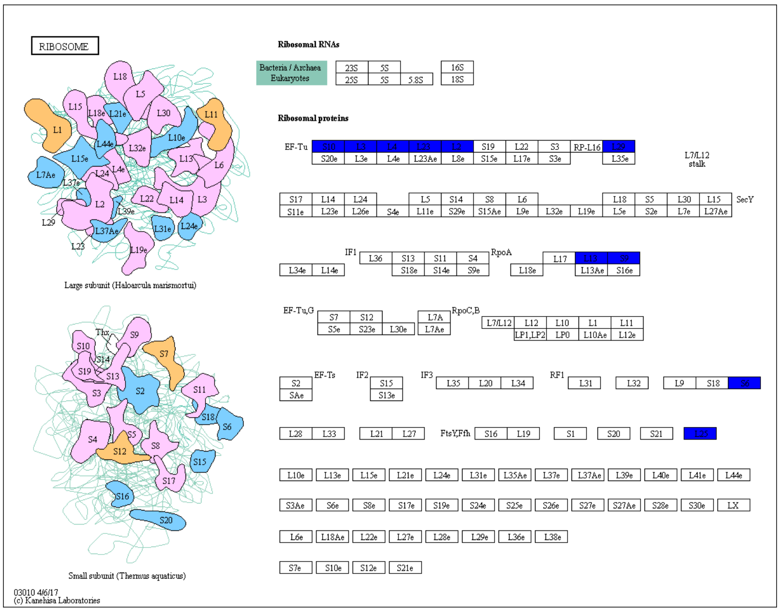


B

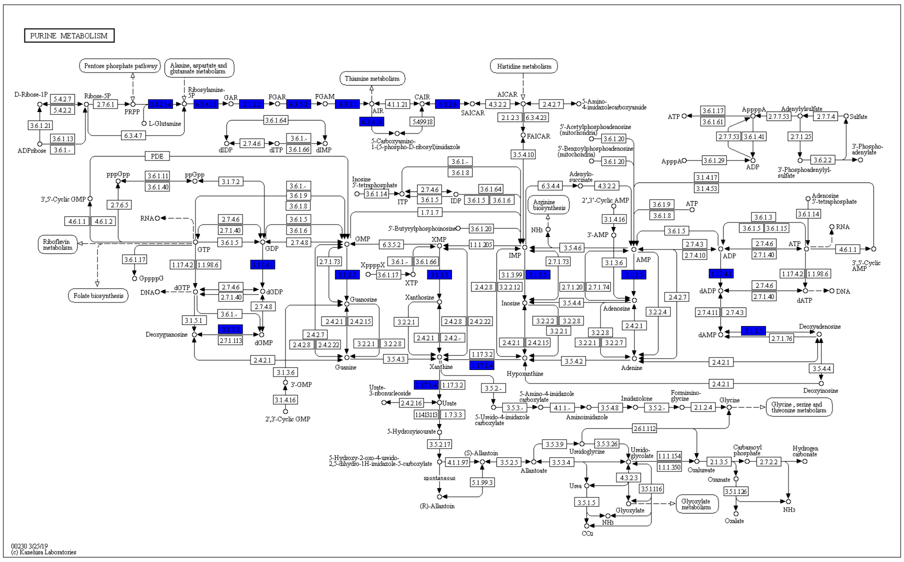


**Supplementary Figure 2.** Significantly enriched KEGG metabolic pathways in *Escherichia coli* strain ECW1 with those in *E. coli* strain ZF43Δ*gdhA*. **(A)** Pathways in ribosome biosynthesis; **(B)** pathways in purine metabolism. The down regulated genes are in blue.

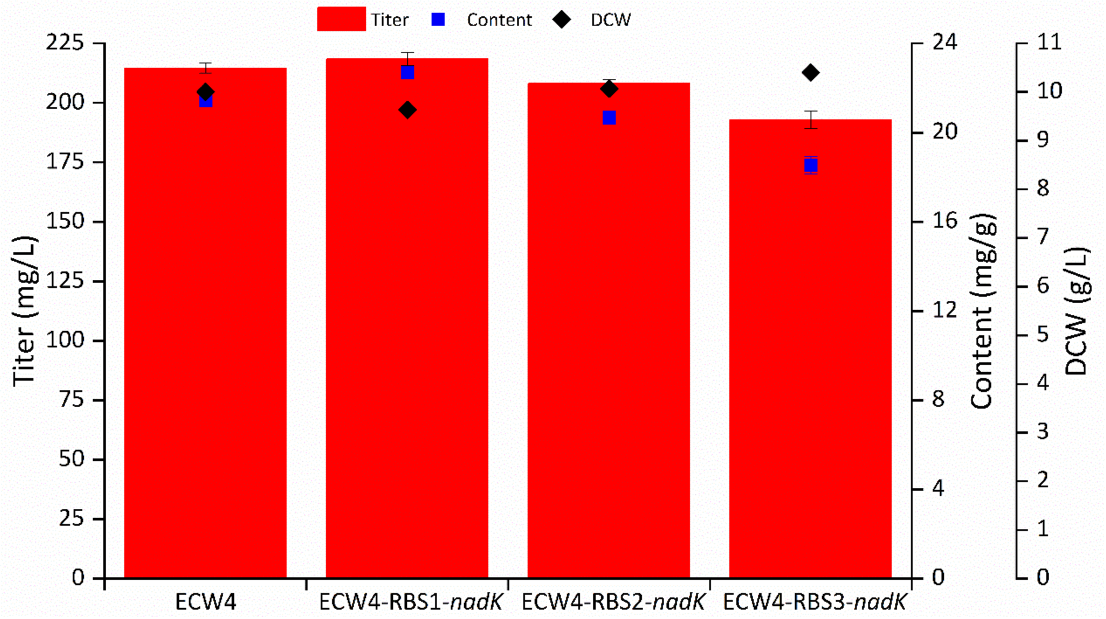


**Supplementary Figure 3.** Effects of ribosome-binding site replacement of *nadK* gene on β-carotene production in *Escherichia coli* strain ECW4. The replacement was carried out using MAGE according to the standard manipulation (Wang et al., 2009). The translation efficiencies of RBS1, RBS2 and RBS3 were increased by 17.98 %, 33.42 % and 46.85 % compared with the wide type RBS of gene *nadK* respectively (Bonde et al., 2016).

**Table S1** *Escherichia coli* strains and plasmids used in this study.

| Strain | Characteristics | Source |
|---|---|---|
| DH5α | $F^-$ ϕ80d*lacZ*ΔM15 Δ(*lacZYAI argF*)*U169 deoR recA1 endA1 hsdR17*($r_K^-$ $m_K^+$) *phoA* Δ*supE44 thi-1 gyrA96 relA1* | Invitrogen |
| ZF43Δ*gdhA* | a β-carotene producing *E. coli* strain derived from EcKan which carries kanamycin resistance gene at the *bioA* loci in its genome | (Li et al., 2015) |
| ECW1 | ZF43Δ*gdhA*, Δ*zwf* | This study |
| ECW2 | ECW1, Δ*ptsH-ptsI-crr* | This study |
| ECW3 | ZF43Δ*gdhA*, Δ*ptsH-ptsI-crr* | This study |
| ECW4 | ECW2, Δ*yjgB* | This study |
| Plasmids | | |
| pTKS/CS | p15A replication, $Cm^r$, $Tet^r$, I-SceI restriction sites | (Cox et al., 2010) |
| pTKRED | pSC101 replication, temperature sensitive replication origin, $Spc^r$, $P_{araBAD}$-driven I-SceI gene, Red recombinase expression plasmid, lac-inducible expression | (Cox et al., 2010) |
| p5C | Expression vector, pSC101 replication, Ptrc, $Amp^r$ | Lab stock |
| p15C | Expression vector, p15A replication, Ptrc, $Amp^r$ | Lab stock |
| p20C | Expression vector, pBR322 replication, Ptrc, $Amp^r$ | Lab stock |
| p15C-pntAB | p15C derivative, expression vector for *pntAB* | This study |
| p15C-sthA | p15C derivative, expression vector for *sthA* | This study |
| p15C-mdh | p15C derivative, expression vector for *mdh* | This study |
| p15C-tPOS5 | p15C derivative, expression vector for *tPOS5* | This study |
| p15C-nadK | p15C derivative, expression vector for *nadK* | This study |
| p5C-nadK | p5C derivative, expression vector for *nadK* | This study |
| p20C-nadK | p20C derivative, expression vector for *nadK* | This study |

**Table S2** Primers used in this study

| Name | Sequences |
|---|---|
| Genes *zwf*, *ptsHIcrr* and *yjgB* deletion | |
| zwf1 | CTGGCTTAAGTACCGGGTTAGTTAACTTAAGGAGAATGACTAG GGATAACAGGGTAATATTTACG |
| zwf2 | GGATAAGCGCAGATATTACTCAAACTCATTCCAGGAACGGTCA TTCTCCTTAAGTTAACTAACCCGGTAATTACCCTGTTATCCCTA CTAAG |
| PTS1 | CCACAACACTAAACCTATAAGTTGGGGAAATACAATGTTCCA GCTAGGGATAACAGGGTAATATTTACG |
| PTS2 | TTTTCACTGCGGCAAGAATTACTTCTTGATGCGGATAACCGCT GGAACATTGTATTTCCCCAACTTATAGATTACCCTGTTATCCCT ACTAAG |
| yjgB1 | GTCGGCAGGCTGTGCTGGCGATACGACAAAACAGAATATGTG CGAAAGAGGGCAGCGCCTCAGATCAGCGCTGCGAATGATTTT AGGGATAACAGGGTAATATTTACG |
| yjgB2 | TTCAGCATTGCATACAGCGATGTGTAACCTTTGTCACACTCCA GGCACCCCGCCCTGCCAAATCATTCGCAGCGCTGATCTGAGG CGCTATTACCCTGTTATCCCTACTAAG |
| Genes *pntAB*, *sthA*, *mdh*, *nadK* and *tPOS5* over-expression | |
| pntA-F | G*GAGCTC*TTGACATCGCATCTTTTTGTACCCATAATTATTTCAT GCGTCATCTAGCATAGGAGGTTTTATGCGAATTGGCATACCAA GAG |
| pntB-R | TCT*AAGCTT*TTACAGAGCTTTCAGGATTGCATC |
| sthA-F | G*GAGCTC*TTGACATCGCATCTTTTTGTACCCATAATTATTTCAT GCGTCATCTAGCATAGGAGGTTTTATGCCACATTCCTACGATTA CG |
| sthA-R | TCT*AAGCTT*TTAAAACAGGCGGTTTAAACCGT |
| mdh-F | G*GAGCTC*TTGACATCGCATCTTTTTGTACCCATAATTATTTCAT GCGTCATCTAGCATAGGAGGTTTTATGAAAGTCGCAGTCCTCG G |
| mdh-R | TCT*AAGCTT*TTACTTATTAACGAACTCTTCGCCCAGG |
| nadK-F | G*GAGCTC*TTGACATCGCATCTTTTTGTACCCATAATTATTTCAT GCGTCATCTAGCATAGGAGGTTTTATGAATAATCATTTCAAGT G |
| nadK-R | TCT*AAGCTT*TTAGAATAATTTTTTGACCAGCCGAGC |
| tPOS5-F | G*GAGCTC*TTGACATCGCATCTTTTTGTACCCATAATTATTTCAT GCGTCATCTAGCATAGGAGGTTTTATGAGTACGTTGGATTCAC |
| tPOS5-R | TCT*AAGCTT*TTAATCATTATCAGTCTGTCTCTTGGTCAGCCT |
| The sequences of RBSs for gene *nadK* regulation | |
| RBS1 | G*G*G*T*TTCAAGGGAAGCACTCACATTGTCATCAATCTTCGC AAcaggggCCTCGGAAAAATGAATAATCATTTCAAGTGTATTGGC ATTGT |
| RBS2 | G*G*G*T*TTCAAGGGAAGCACTCACATTGTCATCAATCTTCGC |

| | |
|---|---|
| | AAaggaacCCTCGGAAAAATGAATAATCATTTCAAGTGTATTGGC ATTGT |
| RBS3 | G*G*G*T*TTCAAGGGAAGCACTCACATTGTCATCAATCTTCGC AAcggaggCCTCGGAAAAATGAATAATCATTTCAAGTGTATTGGC ATTGT |

Duplication regions were in red; The sites of homing endonuclease I-SceI recognition sites sequences were in blue; Promoter apFAB72 and RBS apFAB848 were in pink and double underlines.

**Table S3** Relative expression strengths of genes in *Escherichia coli* strains ECW1 and ECW2. The values in first three columns were the TPM (transcripts per million base pair) values of genes. The ratios represented the relative expression strengths of genes in strains. Ratio-1 = $TPM_{ECW1}/TPM_{ZF43\Delta gdhA}$, ratio-2 = $TPM_{ECW2}/TPM_{ZF43\Delta gdhA}$. Samples of the three strains were sampled at 12 hours.

| | ZF43Δ*gdhA* | ECW1 | ECW2 | Ratio-1 | Ratio-2 |
|---|---|---|---|---|---|
| *dxs* | 1.886268 | 1.019189 | 0.580207 | 0.54032 | 0.307595 |
| *dxr* | 0.016594 | 0.010607 | 0.010291 | 0.639177 | 0.620164 |
| *ispD* | 0.001853 | 0.001561 | 0.000661 | 0.842457 | 0.356719 |
| *ispE* | 0.981098 | 0.610881 | 1.625281 | 0.62265 | 1.656594 |
| *ispF* | 0.001346 | 0.001312 | 0.000459 | 0.974472 | 0.34101 |
| *ispG* | 0.01796 | 0.019795 | 0.022989 | 1.102173 | 1.280011 |
| *ispH* | 0.364455 | 0.316908 | 0.492936 | 0.869541 | 1.352529 |
| *idi* | 0.152494 | 0.147111 | 0.181717 | 0.964698 | 1.191634 |
| *ispA* | 0.488213 | 0.371712 | 0.577673 | 0.761373 | 1.18324 |
| *gps* | 0.284458 | 0.336695 | 0.314822 | 1.183639 | 1.106743 |
| *crtE* | 0.027732 | 0.031763 | 0.011601 | 1.145363 | 0.418325 |
| *crtB* | 0.022377 | 0.024223 | 0.008377 | 1.082534 | 0.374358 |
| *crtI* | 0.020145 | 0.022785 | 0.007498 | 1.131049 | 0.372202 |
| *crtY* | 0.645289 | 0.584534 | 0.657701 | 0.905848 | 1.019235 |

Bonde, M.T., Pedersen, M., Klausen, M.S., Jensen, S.I., Wulff, T., Harrison, S., Nielsen, A.T., Herrgård, M.J., and Sommer, M.O.A. (2016). Predictable tuning of protein expression in bacteria. *Nat. Methods* 13**,** 233.

Cox, E.C., and Kuhlman, T.E. (2010). Site-specific chromosomal integration of large synthetic constructs. *Nucleic Acids Res.* 38**,** e92-e92.

Li, Y., Lin, Z., Huang, C., Zhang, Y., Wang, Z., Tang, Y.-J., Chen, T., and Zhao, X. (2015). Metabolic engineering of *Escherichia coli* using CRISPR–Cas9 meditated genome editing. *Metab. Eng.* 31**,** 13-21.

Wang, H.H., Isaacs, F.J., Carr, P.A., Sun, Z.Z., Xu, G., Forest, C.R., and Church, G.M. (2009). Programming cells by multiplex genome engineering and accelerated evolution. *Nature* 460**,** 894.
